# Hospital mortality from covid-19 in children and adolescents in Brazil in 2020–2021

**DOI:** 10.11606/s1518-8787.2023057005172

**Published:** 2023-09-14

**Authors:** Amanda Cilene Cruz Aguiar Castilho da Silva, Ronir Raggio Luiz, José Rodrigo de Moraes, Pedro Henrique Vieira Rocha, Regina Célia Gollner Zeitoune, Arnaldo Prata Barbosa, Jessica Pronestino de Lima Moreira

**Affiliations:** I Universidade Federal do Rio de Janeiro Escola de Enfermagem Anna Nery Rio de Janeiro RJ Brazil Universidade Federal do Rio de Janeiro. Escola de Enfermagem Anna Nery. Rio de Janeiro, RJ, Brazil.; II Universidade Federal do Rio de Janeiro Instituto de Saúde Coletiva Rio de Janeiro RJ Brazil Universidade Federal do Rio de Janeiro. Instituto de Saúde Coletiva. Rio de Janeiro, RJ, Brazil.; III Universidade Federal Fluminense Instituto de Matemática e Estatística Departamento de Estatística Niterói RJ Brazil Universidade Federal Fluminense. Instituto de Matemática e Estatística. Departamento de Estatística. Niterói, RJ, Brazil.; IV Universidade Federal Fluminense Faculdade de Farmácia Niterói RJ Brazil Universidade Federal Fluminense. Faculdade de Farmácia. Niterói, RJ, Brazil.; V Instituto D’Or de Pesquisa e Ensino Departamento de Pediatria Rio de Janeiro RJ Brazil Instituto D’Or de Pesquisa e Ensino. Departamento de Pediatria. Rio de Janeiro, RJ, Brazil.; VI Universidade Federal Fluminense Faculdade de Farmácia Departamento de Bromatologia Niterói RJ Brazil Universidade Federal Fluminense. Faculdade de Farmácia. Departamento de Bromatologia. Niterói, RJ, Brazil.

**Keywords:** COVID-19, epidemiology, Severe Acute Respiratory Syndrome, Child, Hospital Mortality

## Abstract

**OBJECTIVE:**

To describe cases, deaths, and hospital mortality from covid-19 in children and adolescents in Brazil, according to age group, during the evolving phases of the pandemic in 2020 and 2021.

**METHODS:**

Census of patients aged up to 19 committed with severe acute respiratory syndrome, due to covid-19 or unspecified, notified to the Brazilian Influenza Epidemiological Surveillance Information System, from January 1, 2020, to December 31, 2021. The two years were divided into six phases, covering the spread of the disease—first, second and third wave—as well as the impact of vaccination. The pediatric population was categorized into infants, preschoolers, schoolchildren, and adolescents. Hospital mortality was assessed by pandemic phase and age group.

**RESULTS:**

A total of 144,041 patients were recorded in the two years, 18.2% of whom had confirmed cases of covid-19. Children under 5 years old (infants and preschoolers) accounted for 62.8% of those hospitalized. A total of 4,471 patients died, representing about 6.1 deaths per day. Infants were the ones who most progressed to the intensive care unit (24.7%) and had the highest gross number of deaths (n = 2,012), but mortality was higher among adolescents (5.7%), reaching 9.8% in phase 1. The first peak of deaths occurred in phase 1 (May/2020), and two other peaks occurred in phase 4 (March/2021 and May/2021). There was an increase in cases and deaths for younger ages since phase 4. Hospital mortality in the pediatric population was higher in phases 1, 4, and 6, following the phenomena of dissemination/interiorization of the virus in the country, beginning of the second wave and beginning of the third wave, respectively.

**CONCLUSION:**

The absolute number of cases of covid-19 in children and adolescents is significant. Although complete vaccination in descending order of age provided a natural deviation in age range, there was a greater gap between the curve of new hospitalized cases and the curve of deaths, indicating the positive impact of immunization.

## INTRODUCTION

Covid-19, an infectious respiratory disease caused by a new type of coronavirus that emerged at the end of 2019 in China, caused the most serious epidemic ever experienced in the world in this century^1^. With high transmission power, it quickly spread to neighboring countries and crossed continents, starting with Asia, Europe, and the Americas. In March 2020, the World Health Organization (WHO) declared the new disease a pandemic^2^.

Since the emergence of the disease to September 17, 2022, 611,621,334 cases have been reported and 6,525,419 lives have been lost worldwide. Over two years of the pandemic, Brazil came to be considered second in total accumulated cases in a period of 2020 and in another of 2021. In September 2022, the country ranked fourth in terms of total number of accumulated cases, behind only the United States (USA), India, and France. Regarding deaths, it ranked second from May 2020 to October 2022, with 685,334 deaths from covid-19 in the Brazilian territory, outnumbered only by the USA^3^.

With the follow-up of the first cases, it was highlighted that the pediatric population, despite developing the disease, presented milder conditions as compared to adults^4,5^. However, it was observed that children and adolescents are not free from presenting severe and lethal forms of the disease, such as multisystem inflammatory syndrome in children (MIS-C) and severe acute respiratory syndrome (SARS)^6^.

SARS is the development of two or more respiratory symptoms that characterize the flu-like syndrome (FS), associated with the following signs/symptoms, which indicate severity: dyspnea/respiratory discomfort or pressure or persistent chest pain or O_2_ saturation less than 95% in room air or bluish discoloration (cyanosis) of the lips or face, as defined in the SARS notification form, updated from the Severe Acute Respiratory Syndrome Epidemiological Surveillance Guide (2021)^7^. It is one of the main causes of morbidity and mortality in the pediatric population, often requiring hospitalization in the pediatric intensive care unit (PICU), thus configuring an important problem for world public health^8^.

SARS cases in the pediatric population increased by 34.38% in 2020 compared to 2019. Furthermore, the number of ICU admissions increased by 25.98% and the number of deaths from SARS grew by 65.91%; 70% of cases were classified as unspecified SARS^9^. This definition is used when the etiological agent was not identified in the laboratory test, it was not feasible to collect/process a clinical sample for laboratory diagnosis, or if it was not possible to confirm the diagnosis by clinical-epidemiological or clinical-imaging criteria. Given the increase in the number of cases of unspecified SARS in children and individuals aged up to 19 years, it was considered that these data should not be disregarded, as there is no other justification for this significant increase other than the covid-19 pandemic itself, still in effect.

Although children and adolescents are analyzed as a large age group of individuals up to 19 years old, it is known that there are significant differences in the pattern of illness and death in the age groups of this segment. For a more assertive understanding of the epidemiological situation of children and adolescents in Brazil during the covid-19 pandemic, this study adopted the classification used by the Brazilian Society of Pediatrics^10^, in which the pediatric population is categorized into infants (under 2 years of age), preschoolers (2–4 years of age), school children (5–9 years of age), and teenagers (10–19 years of age). Given that vaccination against covid-19 occurred in a decreasing age pattern, being detrimental to younger people, it is extremely important to assess the form of involvement in the population under 19, stratifying by age groups. According to the Fiocruz Observatory Bulletin^1^, there was a proportional shift of the disease towards younger ages, since infants, preschoolers and part of the school population were not covered by vaccination until the end of 2021.

Throughout 2020 and 2021, changes were observed in the pattern of illness and mortality from the disease. In order to understand the temporal evolution of covid-19 in Brazil and its impact on mortality in children and adolescents, the first two years of the pandemic were divided into six phases related to its main milestones, covering the disease dissemination phase—first, second and third waves—as well as the impact of vaccination initiated in 2021, as suggested by the Fiocruz Observatory Bulletin^1^.

The division into phases is a way of considering the specificity of each moment for a proper understanding of the covid-19 temporal evolution in Brazil.

Since the issue of covid-19 severity in children and adolescents is still open, this study aims to describe the covid-19 cases, deaths and hospital mortality in children and adolescents in Brazil, according to age groups, during the phases of the pandemic in 2020 and 2021.

## METHODS

Census of notified SARS cases from covid-19 and unspecified SARS in individuals up to 19 years old, available in the public database of the Influenza Epidemiological Surveillance Information System (*Sistema de Informação de Vigilância Epidemiológica da Gripe* – Sivep-Gripe), in Brazil. Sivep-Gripe is the official SARS surveillance system and its data come from the compulsory notification forms of hospitalized SARS cases or SARS deaths throughout the country, including public, private and philanthropic establishments. Since the Ministry of Health included SARS-CoV-2 among the etiological agents that cause SARS in 2020, Sivep-Gripe is the official system for reporting and monitoring hospitalizations and deaths of severe cases of covid-19^7^.

In order to build the database, the 2020 and 2021 files of the SARS cases notified in the Sivep-Gripe were downloaded, made available by the Ministry of Health Department of Information Technology^11^.

Next, SARS cases that received the final classification of SARS from covid-19 and unspecified SARS were selected. In order to meet the SARS case definition, we included all hospitalized cases with cough or sore throat accompanied by dyspnea or respiratory distress or saturation lower than 95%, or that evolved to direct death from SARS, regardless of hospitalization. Cases that did not meet the SARS definition, despite being present in Sivep-Gripe, were excluded.

Subsequently, cases with hospitalization or death in 2020 and 2021 in individuals aged 19 or under were selected, totaling 144,041 children and adolescents.

The following inclusion criteria were adopted: children and adolescents from 0 to 19 years of age who were hospitalized and/or died due to SARS, whose final classification was SARS from covid-19 or unspecified SARS, notified to Sivep-Gripe from January 2020 to December 31, 2021. Exclusion criteria were: children and adolescents aged 0 to 19 years, notified in Sivep-Gripe as SARS from covid-19 or unspecified SARS, whose variable “outcome” included “death from other causes,” and the variable “hospitalization” presented the answer “not hospitalized,” provided that the individual had not evolved to death.

The months of 2020 and 2021 were divided into six phases, in which the main milestones of each period are presented, as proposed in the Observatory Bulletin^1^.

Phase 1: January 2020 to May 2020. It comprises the period of SARS-CoV-2 introduction in the national territory and covid-19’s movement of expansion from the capitals to the interior.

Phase 2: June 2020 to August 2020. Period comprised by the first wave of covid-19 and stabilization of transmission indicators, hospitalizations, and deaths at very high values.

Phase 3: September 2020 to November 2020. Interval between the first and second covid-19 waves. Transmission, hospitalization, and death indicators decreased.

Phase 4: December 2020 to June 2021. Second covid-19 wave and further increase in transmission, hospitalization and death indicators. Start of the vaccination campaign in January 2021, prioritizing risk groups listed by the National Vaccination Operational Plan against covid-19. In the late May 2021, vaccination of the general population aged 18 to 59 years began, carried out in a decreasing manner by age, not occurring homogeneously in the national territory.

Phase 5: July 2021 to November 2021. Positive impacts of the vaccination campaign with a reduction in transmission, hospitalization and death indicators, with consequent relief for the health system. Start of vaccination for those over 12 years old in September.

Phase 6: December 2021. Beginning of the third wave. Phase 6 covers a period beyond December 2021, not included here.

In this study, we analyzed the following variables: outcome (death or discharge), age, age group, pandemic phase, month/year of notification, date of hospitalization, date of death, need for ICU, date of ICU admission, final classification of the case, and hospital mortality.

The studied variables were submitted to descriptive statistical analysis to carry out an exploratory data analysis, whose results are displayed in graphs and tables showing the hospital mortality indicator (reason of deaths that occurred among hospitalized patients).

The software used were: IBM SPSS, version 24.0, for data analysis; R, version 4.2.1, for building graphs; Microsoft Office Excel, version 2016, for elaborating the tables.

## RESULTS

Table shows the distribution of SARS hospitalizations, proportion of confirmed covid-19 cases, patients hospitalized in ICU, deaths, and hospital mortality, according to the age groups of children and adolescents during the covid-19 pandemic in Brazil in 2020 and 2021. Infants accounted for 37% of those hospitalized in 2020 and 44.7% in 2021, followed by adolescents. Hospitalized adolescents were the ones who most received the final classification of SARS from covid-19, reaching 40% in 2021. Infants and adolescents progressed more to the ICU, totaling more than 20% of hospitalized patients. In 2020 and 2021, 4,471 deaths occurred, representing about 6.1 deaths/day, referring to hospitalized patients. In 2020, there were 7.7 deaths/day and, in 2021, 4.6 deaths/day. Hospital mortality was higher in 2020 (3.9%) with emphasis on adolescents, with 6%, followed by infants, with 4.8%.

**Table:** Distribution of SARS hospitalizations, proportion of confirmed covid-19 cases, patients hospitalized in ICU, deaths, and hospital mortality, according to age groups of children and adolescents, during the covid-19 pandemic in Brazil, 2020–2021.

Age group	Hospitalized		Covid-19 proportion (%)	Admission to ICU		Deaths	Hospital mortality (%)
	
	n	%		n	%		
2020							
Children and adolescents	71,056	100	18.9	16,280	22.9	2,793	3.9
Infant	26,274	37	17.6	7,334	27.9	1,271	4.8
Preschooler	14,355	20.2	13.7	2,630	18.3	262	1.8
Schoolchildren	13,551	19.1	15	2,569	19	246	1.8
Adolescent	16,876	23.8	28.7	3,747	22.2	1,014	6.0
2021							
Children and adolescents	72,985	100	17.6	14,369	19.7	1,678	2.3
Infant	32,595	44.7	13.8	7,214	22.1	741	2.3
Preschooler	17,249	23.6	10.9	2,610	15.1	142	0.8
Schoolchildren	10,962	15	14.5	1,868	17	145	1.3
Adolescent	12,179	16.7	40	2,677	22	650	5.3
2020–2021							
Children and adolescents	144,041	100	18.2	30,649	21.3	4,471	3.1
Infant	58,869	40.9	15.5	14,548	24.7	2,012	3.4
Preschooler	31,604	21.9	12.2	5,240	16.6	404	1.3
Schoolchildren	24,513	17	14.8	4,437	18.1	391	1.6
Adolescent	29,055	20.2	33.4	6,424	22.1	1,664	5.7

Note: Infants: children under 2 years old; Preschoolers: children aged 2–4 years; Schoolchildren: children aged 5–9 years. Adolescents: individuals aged 10–19 years.

Source: Sivep Gripe/Datasus/Ministry of Health.

**Figure 1:**
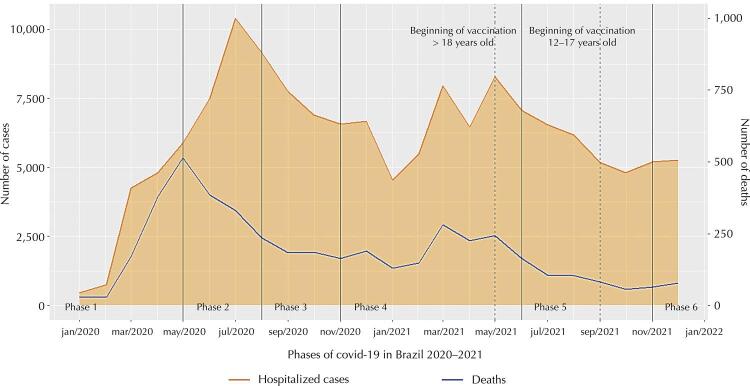
SARS hospitalizations and deaths due to covid-19 and unspecified in children and adolescents (0 to 19 years old) in Brazil 2020 and 2021. Brazil, 2022.

Figure 1 shows SARS hospitalizations and deaths from covid-19 and unspecified SARS in children and adolescents, aged 0 to 19 years, in Brazil, by month, in 2020 and 2021. An exponential increase in cases was observed in phases 1 and 2, with a peak in phase 2, and about 10 thousand new cases in July 2020. There was a decrease in phase 3, an increase in phase 4, with 2 peaks and about 8 thousand new cases in March and May 2021, followed by a decrease in phase 5 and new increase in phase 6. As for the number of deaths, there was an exponential increase in phase 1 and peak, with about 500 deaths in May 2020; drop in phases 2 and 3; increase in phase 4, with 2 peaks, with approximately 275 deaths in March and 250 in May 2021; drop in phase 5; and a new increase in phase 6. The number of deaths and new hospitalizations decreased from June 2021 to October 2021.

SARS: severe acute respiratory syndrome.

Source: Sivep Gripe/Datasus/Ministry of Health.

**Figure 2:**
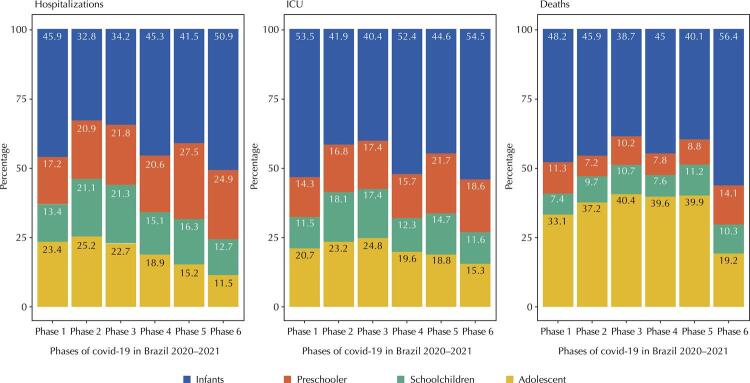
Proportional distribution of SARS hospitalizations, ICU admissions, and deaths of children and adolescents, according to age groups and phases of the pandemic. Brazil: 2020–2021

Figure 2 shows the proportional distribution of SARS hospitalizations from covid-19, ICU admissions, and deaths of children and adolescents, according to age groups and pandemic phases in 2020 and 2021 in Brazil. It can be seen, in relation to hospitalizations, that infants were the most affected in all 6 phases, reaching 50.9% in phase 6. Adolescents went from 23.4% in phase 1 to 11.5 % in phase 6. From phase 4 to phase 6, preschoolers ranked second, having increased by 44.8% from January 2020 to December 2021, that is, from phase 1 to phase 6.

Note 1: Phase 1: January/20 to May/20; Phase 2: June/20 to August/20; Phase 3: September/20 to November/20; Phase 4: December/20 to June/21; Phase 5: July/21 to November/21; and Phase 6: December/21.

Note 2: Infants: children under 2 years old; Preschoolers: children aged 2–4 years; Schoolchild: children aged 5–9 years. Adolescents: individuals aged 10–19 years.

SARS: severe acute respiratory syndrome.

Source: Sivep Gripe/Datasus/Ministry of Health.

Infants accounted for more than half of those admitted to ICU in phases 1, 4, and 6, corresponding to 54.5% in December 2021. Next were the adolescents, up to phase 4, outnumbered by preschoolers in the last phases. Adolescents reduced their representation in ICU admissions by 26.1% during the 2 years studied, while preschoolers increased by 30.1%. Regarding deaths, there is a predominance of the age group of infants, followed by adolescents in all phases. In phase 6, infants accounted for 56.4% of deaths, with a reduction in the participation of all other age groups, with 19.2% of adolescents, 14.1% of preschoolers, and 10.3% of schoolchildren.

**Figure 3:**
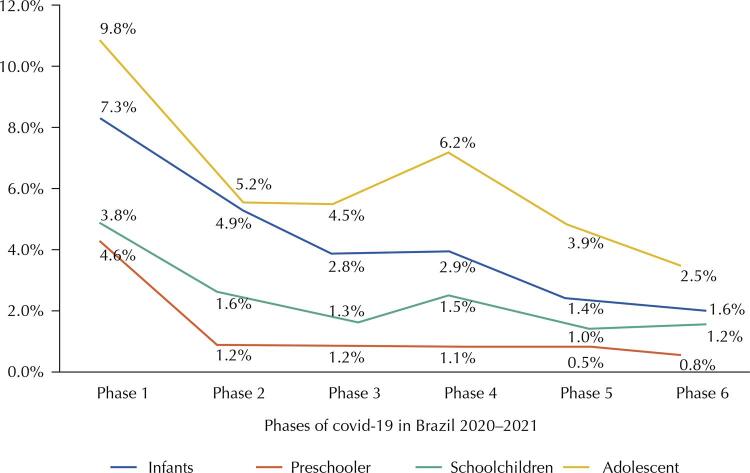
Hospital mortality from SARS due to covid-19 by age groups of children and adolescents in Brazil, according to the phases of the pandemic in 2020–2021. Brazil, 2022.

Figure 3 shows hospital mortality from SARS due to covid-19 among children and adolescents in Brazil by age group, according to the pandemic phases in 2020–2021. Age groups corresponding to adolescents and infants showed higher hospital mortality in all these stages. However, a sharp drop is observed, mainly in these two age groups from phase 5 onwards, coinciding with the beginning of vaccination among adolescents in Brazil. Phase 1 presented the worst hospital mortality scenario for children and adolescents in Brazil.

Note 1: Phase 1: January/20 to May/20; Phase 2: June/20 to August/20; Phase 3: September/20 to November/20; Phase 4: December/20 to June/21; Phase 5: July/21 to November/21; and Phase 6: December/21.

Note 2: Infants: children under 2 years old; Preschoolers: children aged 2–4 years; Schoolchildren: children aged 5–9 years. Adolescents: individuals aged 10–19 years.

Source: Sivep Gripe/Datasus/Ministry of Health.

## DISCUSSION

It was observed that deaths and the hospital mortality rate due to covid-19 among Brazilian children and adolescents occurred differently over the six phases of the pandemic and between the age groups studied. Higher hospital mortality rates were found among adolescents, followed by infants, schoolchildren, and preschoolers. This relationship was maintained throughout all phases. However, the magnitude of the hospital mortality rate was quite high in phase 1.

As vaccination evolved, including ages in a decreasing way, the proportional representation of infants increased in relation to the total number of hospitalizations, to those admitted to ICU and among deaths, mainly from phase 6 onwards. The introduction of vaccination, at the end of May 2021, for adolescents over 18 years of age, and, in September, for the 12-17 age group, entailed a greater distance between the curve of new hospitalized cases and the curve of deaths, indicating the positive impact of vaccination.

Complete vaccination of adults led to a natural shift in age group, proportionally increasing covid-19 cases in the pediatric population^12^. Data from the Fiocruz News Agency indicate that, as of July 2022, for every five people hospitalized due to covid-19 in Brazil, two correspond to children under 5 years of age^13^.

In Brazil, a very high hospital mortality rate was identified since the beginning of the pandemic, reaching 9.8% for adolescents in phase 1. With these data, it is difficult to explain the reasons for such a high rate. Some possibilities should be considered:

1. The presence of comorbidities in this group of patients: a recent study describes that the presence of comorbidities increased by 5.5 times the chance of a more severe disease, represented by the need for mechanical ventilation^14^;
2. Early in the pandemic, the mainstream media and the WHO advised people to maintain home quarantine in asymptomatic cases^2^, causing hospital care to be reserved for cases with more severe symptoms of the disease;
3. Low initial knowledge of the disease and the best form of treatment, especially related to ventilatory support, in which many routines, at the time, advised against the use of non-invasive ventilation due to the risk of generating aerosols, increasing the possibility of virus dissemination, if total isolation of the patient was not possible^2^.

This mortality decreased over time in all age groups, after the change in the Ministry of Health’s guidance on seeking early medical care, especially in the presence of risk factors, and greater knowledge about the disease and the way to act in severe cases.

Studies with the pediatric population carried out in other countries showed a lower mortality rate than that found in Brazil. In the United Kingdom, for example, only 1% of hospitalized children and adolescents with covid-19 died^17^.

A cross-sectional study using data from children and adolescents hospitalized in ICUs in the US and Canada, from March to April 2020, showed a mortality rate of 2% in the group whose median age was 13 years, and the range was 4–16 years^18^. The high mortality rate in a population of children and adolescents in Brazil was reported in a systematic review study that found the worst pediatric mortality rate among 138 countries studied^19^.

A study carried out in Brazil, with notification data of children and adolescents with covid-19 until January 2021, found a rate of 7.3% for hospitalized patients under 20 years of age^20^.

In a meta-analysis carried out from articles published from January to October 2020, a lethality rate of 0.28% was found for the pediatric population^21^. In the United Kingdom, another study, when investigating the community prevalence of covid-19, found a lethality rate of less than 0.5% in the children and adolescents group in the period between the beginning of the pandemic and the end of the first wave^22^.

In this study, we chose to use the hospital mortality indicator, given that low testing makes it impossible to calculate lethality, which deals with the proportion of deaths among all patients. A study on testing capacity in Brazil found that it was low compared to other countries and considered insufficient^23^. Infants and adolescents stood out, with higher mortality among patients younger than 2 years old and older than 12 years old^20^.

In Europe, another study found a higher percentage of the severe form of covid-19 among hospitalized patients under 2 years old and over 10 years old^24^. In the USA, a study with individuals under 21 years old with SARS due to covid-19, hospitalized from February to July 2020, found a higher mortality rate among adolescents^25^. A meta-analysis and systematic review carried out from January to October 2020, with hospitalized children who required ventilatory support or ICU as subjects, also pointed out infants and adolescents as the groups with the highest hospital mortality in pediatrics^26^.

A study carried out in an ICU in Brazil, from March to May 2020, found a mortality of 3% in subjects aged between 1 month and 19 years, median age of 4 years, 25% of patients being infants, and 44% children in school age, adolescents, and young adults^14^.

One of the strengths of this research is the use of clinical and demographic data available in Sivep-Gripe through the compulsory notification of SARS cases, allowing to carry out a census of the hospitalized pediatric population.

The limitations of this research include the possibility of underreporting of cases, delays in notifying Sivep-Gripe, and the hacker attack on the Sivep-Gripe database in December 2021, which may result in information bias. It is likely that the numbers are even higher than the already significant data reported here.

We conclude that the absolute number of cases of covid-19 in children and adolescents is significant and posed a serious health problem for the country in 2020 and 2021, due to the high rates of hospitalization and hospital mortality in that period, In 2020, 7.7 individuals from the group of children and adolescents died per day, falling to 4.6 individuals in 2021, which corresponds to 6.1 deaths per day among individuals up to 19 years of age hospitalized with SARS in 2020 and 2021.

The highest hospital mortality rate was associated with infants and adolescents, with an increase in cases and deaths for younger ages since phase 4. Vaccination against covid-19 began in phase 4 for those over 18 years of age and, in phase 5, for those over 12, showing a strong impact on the reduction of hospitalizations and deaths in the pediatric population.

The hospital mortality rate in the pediatric population in Brazil was higher in phases 1, 2, and 3, thus accompanying the phenomena of dissemination/interiorization of the virus in the country, beginning of the second wave, and beginning of the third wave, respectively.

The importance of vaccinating preschoolers and infants is emphasized, in view of the approval and release, by Anvisa, for the vaccination of children older than 6 months, on September 16, 2022, in order to minimize the effects of pandemic in the age group of children and adolescents and provide indirect protection for infants younger than 6 months who have not yet been vaccinated.
