# Supplementary material for: Hospital mortality from covid-19 in children and adolescents in Brazil in 2020–2021
Source: Rev Saude Publica. 2023 Sep 14;57:56. doi: 10.11606/s1518-8787.2023057005172 (PMC10519682; doi:10.11606/s1518-8787.2023057005172)
Supplement: Translation

# Translation: Mortalidade hospitalar por covid-19 em crianças e adolescentes no Brasil em 2020–2021

**Authors:** Amanda Cilene Cruz Aguiar Castilho da Silva, Ronir Raggio Luiz, José Rodrigo de Moraes, Pedro Henrique Vieira Rocha, Regina Célia Gollner Zeitoune, Arnaldo Prata Barbosa, Jessica Pronestino de Lima Moreira

**Affiliations:** I Brazil Universidade Federal do Rio de Janeiro. Escola de Enfermagem Anna Nery. Rio de Janeiro, RJ, Brazil.; II Brazil Universidade Federal do Rio de Janeiro. Instituto de Saúde Coletiva. Rio de Janeiro, RJ, Brazil.; III Brazil Universidade Federal Fluminense. Instituto de Matemática e Estatística. Departamento de Estatística. Niterói, RJ, Brazil.; IV Brazil Universidade Federal Fluminense. Faculdade de Farmácia. Niterói, RJ, Brazil.; V Brazil Instituto D’Or de Pesquisa e Ensino. Departamento de Pediatria. Rio de Janeiro, RJ, Brazil.; VI Brazil Universidade Federal Fluminense. Faculdade de Farmácia. Departamento de Bromatologia. Niterói, RJ, Brazil.

**Keywords:** COVID-19, epidemiologia, Síndrome Respiratória Aguda Grave, Criança, Mortalidade Hospitalar

## Abstract

**OBJETIVO:**

Descrever casos, óbitos e mortalidade hospitalar por covid-19 em crianças e adolescentes no Brasil, conforme faixa etária, durante as fases de evolução da pandemia em 2020 e 2021.

**MÉTODOS:**

Censo de pacientes de até 19 anos internados com síndrome respiratória aguda grave, por covid-19 ou não especificada, notificados ao Sistema de Informação de Vigilância Epidemiológica da Gripe do Brasil, entre 1 de janeiro de 2020 e 31 de dezembro de 2021. Os dois anos foram divididos em seis fases, abrangendo a disseminação da doença − primeira, segunda e terceira onda −, bem como o impacto da vacinação. A população pediátrica foi categorizada em lactentes, pré-escolares, escolares e adolescentes. A mortalidade hospitalar foi avaliada por fase da pandemia e faixa etária.

**RESULTADOS:**

Foram contabilizados 144.041 pacientes nos dois anos, sendo 18,2% casos de covid-19 confirmados. Menores de 5 anos (lactentes e pré-escolares) corresponderam a 62,8% dos hospitalizados. Evoluíram a óbito 4.471, representando cerca 6,1 óbitos por dia. Os lactentes foram os que mais evoluíram para unidade de terapia intensiva (24,7%) e apresentaram o maior número bruto de óbito (n = 2.012), porém a mortalidade foi maior entre os adolescentes (5,7%), chegando a 9,8% na fase 1. O primeiro pico de óbitos ocorreu na fase 1 (maio/2020), e outros dois picos ocorreram na fase 4 (março/2021 e maio/2021). Verificou-se avanço de casos e óbitos para as idades inferiores desde a fase 4. A mortalidade hospitalar na população pediátrica foi maior nas fases 1, 4 e 6, acompanhando os fenômenos de disseminação/interiorização do vírus no país, início da segunda onda e início da terceira onda, respectivamente.

**CONCLUSÃO:**

O número absoluto de casos de covid-19 em crianças e adolescentes é expressivo. Embora a vacinação completa em ordem decrescente de idade tenha proporcionado um desvio natural de faixa etária, ocorreu um distanciamento maior entre a curva de novos casos hospitalizados e a curva de óbitos, indicando o impacto positivo da imunização.

## INTRODUÇÃO

A covid-19, doença infecciosa respiratória causada por um novo tipo de coronavírus que surgiu no final de 2019 na China, provocou a mais grave epidemia já vivida no mundo neste século^1^. Com alto poder de transmissão, rapidamente se espalhou pelos países vizinhos e atravessou continentes, começando pela Ásia, Europa e Américas. Em março de 2020, a Organização Mundial da Saúde (OMS) declarou a nova doença como pandemia^2^.

Desde o surgimento da doença até 17 de setembro de 2022, notificaram-se 611.621.334 casos e 6.525.419 vidas foram perdidas em todo o mundo. Ao longo de dois anos da pandemia, o Brasil chegou a ser considerado segundo lugar em total de casos acumulados em um período de 2020 e em outro de 2021. Em setembro de 2022, o país ocupava a quarta posição quanto ao número total de casos acumulados, sendo precedido por Estados Unidos (EUA), Índia e França. Em relação aos óbitos, encontra-se em segundo lugar desde maio de 2020 até outubro de 2022, com 685.334 óbitos por covid-19 no território brasileiro, sendo precedido apenas pelos EUA^3^.

Com o acompanhamento dos primeiros casos, destacou-se o fato de a população pediátrica, apesar de desenvolver a doença, apresentar quadros mais leves quando comparada aos adultos^4,5^. Contudo, observou-se que crianças e adolescentes não estão isentos de apresentar formas graves e letais da doença, como a síndrome inflamatória multissistêmica pediátrica (SIM-P) e a síndrome respiratória aguda grave (SRAG)^6^.

A SRAG é o desenvolvimento de dois sintomas respiratórios ou mais que caracterizam síndrome gripal (SG), associados aos seguintes sinais/sintomas, que indicam gravidade: dispneia/desconforto respiratório ou pressão ou dor persistente no tórax ou saturação de O_2_ menor que 95% em ar ambiente ou coloração azulada (cianose) dos lábios ou rosto, conforme a definição da ficha de notificação de SRAG, atualizada a partir do Guia de Vigilância Epidemiológica da Síndrome Respiratória Aguda Grave (2021)^7^. Trata-se de uma das principais causas de morbidade e mortalidade da população pediátrica, necessitando, muitas vezes, de internação na unidade de terapia intensiva pediátrica (UTIP), configurando, portanto, problema importante para a saúde pública mundial^8^.

Em 2020, em comparação com 2019, houve aumento de 34,38% nos casos de SRAG na população pediátrica. Além disso, o número de admissões em UTI aumentou 25,98% e o de óbitos por SRAG cresceu 65,91%; 70% dos casos foram classificados como SRAG não especificada^9^. Essa definição é utilizada quando não há identificação de agente etiológico no exame laboratorial, não foi viável coletar/processar amostra clínica para diagnóstico laboratorial ou, ainda, se não foi possível confirmar o diagnóstico por critério clínico-epidemiológico ou clínico-imagético. Diante da alta do número de casos de SRAG não especificada em crianças e em indivíduos com até 19 anos, considerou-se que esses dados não deveriam ser desprezados, tendo em vista não haver outra justificativa para esse aumento expressivo, a não ser a própria pandemia de covid-19, ainda vigente.

Embora crianças e adolescentes sejam analisados como um grande grupo etário de indivíduos até 19 anos, sabe-se que existem diferenças significativas no padrão de adoecimento e óbito nas faixas etárias desse segmento. Para uma compreensão mais assertiva da situação epidemiológica de crianças e adolescentes no Brasil durante a pandemia de covid-19, adotou-se, neste estudo, a classificação utilizada pela Sociedade Brasileira de Pediatria^10^, na qual a população pediátrica é categorizada em lactentes (menores de 2 anos), pré-escolares (2 a 4 anos), escolares (5 a 9 anos) e adolescentes (10 a 19 anos). Tendo em vista que a vacinação contra covid-19 ocorreu em um padrão decrescente de idade, não favorecendo os mais jovens, é extremamente importante avaliar a forma de acometimento na população menor de 19 anos, estratificando pelas faixas etárias. Segundo o Boletim Observatório da Fiocruz^1^, houve um deslocamento proporcional da doença para as idades mais jovens, já que lactentes, pré-escolares e parte da população escolar não foram contemplados com a vacinação até o final de 2021.

Ao longo de 2020 e 2021, observaram-se modificações no padrão de adoecimento e mortalidade pela doença. Para compreender a evolução temporal da covid-19 no Brasil e sua repercussão na mortalidade em crianças e adolescentes, os dois primeiros anos da pandemia foram divididos em seis fases relacionadas aos seus principais marcos, abrangendo a fase de disseminação da doença − primeira, segunda e terceira onda −, bem como o impacto da vacinação iniciada em 2021, conforme sugerido pelo Boletim Observatório da Fiocruz.^1^

A divisão em fases é uma forma de considerar a especificidade de cada momento para compreensão adequada da evolução temporal da covid-19 no Brasil.

Em função da questão da gravidade da covid-19 em crianças e adolescentes ainda estar em aberto, este estudo tem como objetivo descrever os casos, óbitos e mortalidade hospitalar por covid-19 em crianças e adolescentes no Brasil, segundo faixas etárias, durante as fases da pandemia em 2020 e 2021.

## MÉTODOS

Censo dos casos notificados de SRAG por covid-19 e SRAG não especificada, em indivíduos de até 19 anos, disponibilizados em banco de dados público do Sistema de Informação de Vigilância Epidemiológica da Gripe (Sivep-Gripe), no Brasil. O Sivep-Gripe constitui o sistema oficial de vigilância da SRAG e seus dados são provenientes das fichas de notificação compulsória de casos de SRAG hospitalizados ou óbitos por SRAG em todo o país, incluindo estabelecimentos públicos, privados e filantrópicos. Desde que o Ministério da Saúde anexou o SARS-CoV-2 entre os agentes etiológicos causadores de SRAG, em 2020, o Sivep-Gripe é o sistema oficial para relatar e monitorar internações e óbitos de casos graves de covid-19 ^7^.

Para a construção do banco de dados, baixaram-se os arquivos de 2020 e 2021 dos casos de SRAG notificados no Sivep-Gripe, disponibilizados pelo Departamento de Informática do Ministério da Saúde^11^.

A seguir, selecionaram-se os casos de SRAG que receberam classificação final de SRAG por covid-19 e SRAG não especificada. A fim de atender a definição de caso de SRAG, incluíram-se todos os casos hospitalizados que apresentassem tosse ou dor de garganta, acompanhados de dispneia ou desconforto respiratório ou saturação menor que 95%, ou evoluíram a óbito direto por SRAG, independentemente de internação. Excluíram-se os casos que não atendiam a definição de SRAG, apesar de estarem presentes no Sivep-Gripe.

Posteriormente, selecionaram-se os casos com hospitalização ou óbito em 2020 e 2021 em indivíduos com idade menor ou igual a 19 anos, totalizando 144.041 crianças e adolescentes.

Adotaram-se como critérios de inclusão: crianças e adolescentes de 0 a 19 anos de idade hospitalizados e/ou óbitos por SRAG, cuja classificação final foi SRAG por covid-19 ou SRAG não especificada, notificados ao Sivep-Gripe no período de janeiro de 2020 a 31 de dezembro de 2021. Os critérios de exclusão foram: crianças e adolescentes de 0 a 19 anos, notificados no Sivep-Gripe como SRAG por covid-19 ou SRAG não especificada, cuja variável “Evolução” constasse “óbito por outras causas”, e a variável “hospitalização” apresentasse a resposta “não hospitalizado”, desde que o indivíduo não tivesse evoluído a óbito.

Os meses de 2020 e 2021 foram divididos em seis fases, nas quais são apresentados os principais marcos de cada período, conforme proposto no Boletim Observatório^1^.

Fase 1: janeiro de 2020 a maio de 2020. Compreende o período de introdução do SARS-CoV-2 em território nacional e o movimento de expansão da covid-19 das capitais ao interior.

Fase 2: junho de 2020 a agosto de 2020. Período compreendido pela primeira onda da covid-19 e estabilização dos indicadores de transmissão, hospitalizações e óbitos em valores muito elevados.

Fase 3: setembro de 2020 a novembro de 2020. Intervalo entre a primeira e segunda ondas da covid-19. Ocorreram quedas nos indicadores de transmissão, hospitalização e óbito.

Fase 4: dezembro de 2020 a junho de 2021. Segunda onda da covid-19 e novo aumento dos indicadores de transmissão, hospitalização e óbito. Início da campanha de vacinação em janeiro de 2021, priorizando grupos de risco elencados pelo Plano Nacional de Operacionalização de Vacinação contra covid-19. Ao final de maio de 2021, iniciou-se a vacinação da população geral de 18 a 59 anos, realizada de forma decrescente por idade, não ocorrendo de modo homogêneo em território nacional.

Fase 5: julho de 2021 a novembro de 2021. Impactos positivos da campanha vacinal com redução dos indicadores de transmissão, hospitalização e óbito, com consequente alívio do sistema de saúde. Início da vacinação para os maiores de 12 anos em setembro.

Fase 6: dezembro de 2021. Início da terceira onda. A fase 6 percorre um período para além de dezembro de 2021, aqui não incluído.

Neste estudo, foram analisadas as variáveis evolução (óbito ou alta), idade, faixa etária, fase da pandemia, mês/ano de notificação, data de hospitalização, data de óbito, necessidade de UTI, data de entrada na UTI, classificação final do caso, mortalidade hospitalar.

As variáveis foram submetidas à análise estatística descritiva, sendo realizada a análise exploratória dos dados, cujos resultados estão expostos em gráficos e tabelas apresentando o indicador mortalidade hospitalar (razão dos óbitos que ocorreram entre os hospitalizados).

Os softwares utilizados foram: IBM SPSS, versão 24.0, para análise dos dados; R, versão 4.2.1, para construção dos gráficos; Microsoft Office Excel, versão 2016, para elaboração de tabelas.

## RESULTADOS

A Tabela apresenta a distribuição de hospitalizações por SRAG, proporção de casos de covid-19 confirmados, hospitalizados em UTI, óbitos e mortalidade hospitalar, segundo as faixas etárias das crianças e adolescentes durante a pandemia de covid-19 no Brasil em 2020 e 2021. Os lactentes representaram 37% de hospitalizados em 2020 e 44,7% em 2021, seguidos por adolescentes. Os adolescentes hospitalizados foram os que mais receberam classificação final de SRAG por covid-19, chegando a 40% em 2021. Os lactentes e os adolescentes evoluíram mais para UTI, totalizando mais de 20% dos hospitalizados. Em 2020 e 2021, ocorreram 4.471 óbitos, que representam cerca de 6,1 óbitos/dia, referente aos hospitalizados. Em 2020, contabilizaram-se 7,7 óbitos/dia e, em 2021, 4,6 óbitos/dia. A mortalidade hospitalar foi maior em 2020 (3,9%) com destaque para os adolescentes, com 6%, seguidos por lactentes, com 4,8%.

**Tabela:** Distribuição de hospitalizações por SRAG, proporção de covid-19 confirmada, hospitalizados em UTI, óbitos e mortalidade hospitalar, de acordo com as faixas etárias das crianças e adolescentes, durante a pandemia de covid-19 no Brasil, 2020–2021.

Faixa etária	Hospitalizados		Proporção covid-19 (%)	Evolução para UTI		Óbitos	Mortalidade hospitalar (%)
	
	n	%		n	%		
2020							
Crianças e adolescentes	71.056	100	18,9	16.280	22,9	2.793	3,9
Lactente	26.274	37	17,6	7.334	27,9	1.271	4,8
Pré-escolar	14.355	20,2	13,7	2.630	18,3	262	1,8
Escolar	13.551	19,1	15	2.569	19	246	1,8
Adolescente	16.876	23,8	28,7	3.747	22,2	1.014	6,0
2021							
Crianças e adolescentes	72.985	100	17,6	14.369	19,7	1.678	2,3
Lactente	32.595	44,7	13,8	7.214	22,1	741	2,3
Pré-escolar	17.249	23,6	10,9	2.610	15,1	142	0,8
Escolar	10.962	15	14,5	1.868	17	145	1,3
Adolescente	12.179	16,7	40	2.677	22	650	5,3
2020–2021							
Crianças e adolescentes	144.041	100	18,2	30.649	21,3	4.471	3,1
Lactente	58.869	40,9	15,5	14.548	24,7	2.012	3,4
Pré-escolar	31.604	21,9	12,2	5.240	16,6	404	1,3
Escolar	24.513	17	14,8	4.437	18,1	391	1,6
Adolescente	29.055	20,2	33,4	6.424	22,1	1.664	5,7

Nota: Lactentes: crianças menores de 2 anos; Pré-escolares: crianças de 2 a 4 anos; Escolares: crianças de 5 a 9 anos. Adolescentes: indivíduos de 10 a 19 anos.

Fonte: Sivep Gripe/Datasus/Ministério da Saúde.

A Figura 1 apresenta hospitalizações e óbitos por SRAG por covid-19 e SRAG não especificada em crianças e adolescentes, de 0 a 19 anos, no Brasil, por mês, em 2020 e 2021. Observou-se aumento exponencial de casos nas fases 1 e 2, com pico na fase 2 e cerca de 10 mil casos novos em julho de 2020. Ocorreu queda na fase 3, elevação na fase 4, com 2 picos e cerca de 8 mil casos novos em março e maio de 2021, seguida por queda na fase 5 e nova elevação na fase 6. Quanto ao número de óbitos, observou-se: aumento exponencial na fase 1 e pico, com cerca de 500 óbitos em maio de 2020; queda nas fases 2 e 3; elevação na fase 4, com 2 picos, ocorrendo cerca de 275 óbitos em março e 250 em maio de 2021; queda na fase 5; e nova elevação na fase 6. O número de óbitos e novas hospitalizações apresentou queda a partir de junho de 2021 até outubro de 2021.

**Figura 1:** Hospitalizações e óbitos por SRAG por covid-19 e não especificada em crianças e adolescentes (0 a 19 anos) no Brasil 2020 e 2021. Brasil, 2022.

SRAG: síndrome respiratória aguda grave.

Fonte: Sivep Gripe/Datasus/Ministério da Saúde.

A Figura 2 apresenta a distribuição proporcional de hospitalizações de SRAG por covid-19, internações em UTI e óbitos de crianças e adolescentes, conforme faixas etárias e fases da pandemia em 2020 e 2021 no Brasil. Verifica-se, em relação às hospitalizações, que os lactentes foram os mais atingidos em todas as 6 fases, chegando a 50,9% na fase 6. Os adolescentes saíram de 23,4%, na fase 1, para 11,5% na fase 6. Da fase 4 até a fase 6, os pré-escolares ocuparam a segunda posição, tendo aumentado 44,8% de janeiro de 2020 até dezembro de 2021, ou seja, da fase 1 para a fase 6.

**Figura 2:** Distribuição proporcional de hospitalizações de SRAG, internações em UTI e óbitos de crianças e adolescentes, de acordo com as faixas etárias e fases da pandemia. Brasil: 2020–2021

Nota 1: Fase 1: janeiro/20 a maio/20; Fase 2: junho/20 a agosto/20; Fase 3: setembro/20 a novembro/20; Fase 4: dezembro/20 a junho/21; Fase 5: julho/21 a novembro/21; e Fase 6: dezembro/21.

Nota 2: Lactentes: crianças menores de 2 anos; Pré-escolares: crianças de 2 a 4 anos; Escolares: crianças de 5 a 9 anos. Adolescentes: indivíduos de 10 a 19 anos.

SRAG: síndrome respiratória aguda grave; UTI: unidade de terapia intensiva.

Fonte: Sivep Gripe/Datasus/Ministério da Saúde.

Os lactentes foram mais da metade dos internados em UTI nas fases 1, 4 e 6, correspondendo a 54,5% em dezembro de 2021. Em seguida, estavam os adolescentes até a fase 4, superados pelos pré-escolares nas últimas fases. Os adolescentes reduziram 26,1% da sua representação em internações na UTI durante os 2 anos estudados, enquanto os pré-escolares aumentaram 30,1%. Em relação aos óbitos, observa-se predominância da faixa etária de lactentes, seguidos por adolescentes em todas as fases. Na fase 6, os lactentes representaram 56,4% dos óbitos, com redução da participação de todas as outras faixas etárias, com 19,2% dos adolescentes, 14,1% dos pré-escolares e 10,3% dos escolares.

A Figura 3 apresenta a mortalidade hospitalar por SRAG decorrente da covid-19 de crianças e adolescentes no Brasil por faixas etárias, segundo as fases da pandemia em 2020–2021. Faixas etárias correspondentes a adolescentes e a lactentes apresentaram maior mortalidade hospitalar em todas essas fases. Entretanto, observa-se uma queda acentuada, principalmente nessas duas faixas a partir da fase 5, coincidindo com o início da vacinação em adolescentes no Brasil. A fase 1 apresentou o pior cenário de mortalidade hospitalar para crianças e adolescentes no Brasil.

**Figura 3:** Mortalidade hospitalar por SRAG por covid-19 por faixas etárias das crianças e adolescentes no Brasil, segundo as fases da pandemia em 2020–2021. Brasil, 2022.

Nota 1: Fase 1: janeiro/20 a maio/20; Fase 2: junho/20 a agosto/20; Fase 3: setembro/20 a novembro/20; Fase 4: dezembro/20 a junho/21; Fase 5: julho/21 a novembro/21; e Fase 6: dezembro/21.

Nota 2: Lactentes: crianças menores de 2 anos; Pré-escolares: crianças de 2 a 4 anos; Escolares: crianças de 5 a 9 anos. Adolescentes: indivíduos de 10 a 19 anos.

SRAG: síndrome respiratória aguda grave.

Fonte: Sivep Gripe/Datasus/Ministério da Saúde

## DISCUSSÃO

Observou-se que os óbitos e a taxa de mortalidade hospitalar por covid-19 entre crianças e adolescentes brasileiros ocorreram de maneira diversa ao longo das seis fases da pandemia e entre as faixas etárias estudadas. Maiores taxas de mortalidade hospitalar foram encontradas entre os adolescentes, seguidos pelos lactentes, escolares e pré-escolares. Essa relação se manteve por todas as fases. Entretanto, a magnitude da taxa de mortalidade hospitalar foi bastante elevada na fase 1.

Conforme a vacinação foi evoluindo, contemplando as idades de forma decrescente, aumentou a representação proporcional dos lactentes em relação ao total de hospitalizações, aos internados em UTI e entre os óbitos, principalmente a partir da fase 6. A introdução da vacinação, ao final de maio de 2021, para os adolescentes acima de 18 anos e, em setembro, para a faixa de 12 a 17 anos, implicou um distanciamento maior entre a curva de novos casos hospitalizados e a curva de óbitos, indicando o impacto positivo da vacinação.

A vacinação completa dos adultos acarretou um desvio natural de faixa etária, aumentando proporcionalmente os casos de covid-19 na população pediátrica^12^. Dados da Agência de Notícias da Fiocruz apontam que, a partir de julho de 2022, a cada cinco hospitalizados por covid-19, no Brasil, dois correspondem a menores de 5 anos^13^.

No Brasil, identificou-se taxa de mortalidade hospitalar bem elevada desde o início da pandemia, chegando a alcançar 9,8% para os adolescentes na fase 1. Com esses dados, é difícil explicar as razões para essa taxa tão elevada. Algumas possibilidades devem ser consideradas:

1. A presença de comorbidades nesse grupo de pacientes: estudo recente descreve que a presença de comorbidades aumentou em 5,5 vezes a chance de doença mais grave, representada pela necessidade de ventilação mecânica;^14^
2. No início da pandemia, a grande mídia e a OMS orientavam a pessoas a manter quarentena domiciliar em casos assintomáticos^2^, ocasionando a reserva do atendimento hospitalar para casos com sintomas mais graves da doença;
3. O baixo conhecimento inicial da doença e da melhor forma de tratamento, especialmente relacionado ao suporte ventilatório, em que muitas rotinas, na ocasião, desaconselhavam o uso de ventilação não invasiva pelo risco de geração de aerossóis, aumentando a possibilidade de disseminação do vírus, caso não fosse possível o isolamento total do paciente^2^.

Essa mortalidade foi decrescendo ao longo do tempo em todas as faixas etárias, após a mudança de orientação do Ministério da Saúde sobre a procura por atendimento médico precoce, especialmente na presença de fatores de risco, e maior conhecimento sobre a doença e a forma de atuação em casos graves.

Estudos realizados em outros países com a população pediátrica apresentaram taxa de mortalidade menor que a encontrada no Brasil. No Reino Unido, por exemplo apenas 1% das crianças e adolescentes com covid-19 hospitalizados evoluíram para óbito^17^.

Estudo transversal utilizando dados de crianças e adolescentes hospitalizados em UTI nos EUA e no Canadá, no período de março a abril de 2020, mostrou taxa de mortalidade de 2% no grupo cuja mediana de idade foi 13 anos, e a amplitude foi 4–16 anos^18^. A alta taxa de mortalidade em população de crianças e adolescentes no Brasil foi relatada em estudo de revisão sistemática que apontou a pior taxa de mortalidade pediátrica entre 138 países estudados^19^.

Estudo realizado no Brasil, com dados de notificação de crianças e adolescentes com covid-19 até janeiro de 2021, encontrou taxa de 7,3% para menores de 20 anos hospitalizados^20^.

Em metanálise realizada a partir de artigos publicados de janeiro a outubro de 2020, verificou-se taxa de letalidade de 0,28% para população pediátrica^21^. No Reino Unido, outro estudo, ao pesquisar a prevalência comunitária de covid-19, encontrou taxa de letalidade menor que 0,5% no grupo de crianças e adolescentes no período entre o início da pandemia e o final da primeira onda^22^.

Neste estudo, optou-se por utilizar o indicador de mortalidade hospitalar, tendo em vista que a baixa testagem inviabiliza o cálculo da letalidade, que trata da proporção de óbitos entre todos os doentes. Estudo sobre a capacidade de testagem no Brasil constatou que esta foi baixa em comparação a outros países e considerada insuficiente^23^. Destacaram-se os lactentes e adolescentes, sendo a mortalidade maior entre os menores de 2 anos e os maiores de 12 anos^20^.

Na Europa, outro estudo constatou porcentagem maior da forma grave da covid-19 entre os hospitalizados menores de 2 anos e maiores de 10 anos^24^. Nos EUA, estudo com indivíduos menores de 21 anos com SRAG por covid-19, hospitalizados de fevereiro a julho de 2020, encontrou taxa de mortalidade maior entre os adolescentes^25^. Metanálise e revisão sistemática realizadas no período de janeiro a outubro de 2020, tendo como sujeitos crianças hospitalizadas que necessitaram de suporte ventilatório ou UTI, também apontou lactentes e adolescentes como grupos com maior mortalidade hospitalar na pediatria^26^.

Estudo realizado em UTI no Brasil, no período de março a maio de 2020, revelou mortalidade de 3% nos sujeitos com idade entre 1 mês e 19 anos, mediana de idade de 4 anos, sendo 25% dos pacientes lactentes, e 44% crianças em idade escolar, adolescentes e adultos jovens^14^.

Destaca-se, como ponto forte desta pesquisa, o uso dos dados clínicos e demográficos disponíveis no Sivep-Gripe por meio da notificação compulsória de casos de SRAG, permitindo a realização do censo da população pediátrica hospitalizada.

Como limitações desta pesquisa, têm-se a possibilidade de subnotificação de casos, atrasos na notificação ao Sivep-Gripe e o ataque *hacker* ao banco do Sivep-Gripe em dezembro de 2021, que podem resultar em viés de informação. É provável que os números sejam ainda maiores que os já expressivos dados apresentados neste estudo.

Conclui-se que o número absoluto de casos de covid-19 em crianças e adolescentes é expressivo e constituiu um grave problema de saúde ao país, nos anos de 2020 e 2021, devido à alta taxa de hospitalização e mortalidade hospitalar desse período. Foram a óbito 7,7 indivíduos do grupo de crianças e adolescentes por dia em 2020, havendo queda para 4,6 indivíduos em 2021, o que corresponde a 6,1 óbitos por dia, entre os indivíduos de até 19 anos hospitalizados com SRAG em 2020 e 2021.

A maior taxa de mortalidade hospitalar esteve associada a lactentes e a adolescentes, com avanço de casos e óbitos para as idades inferiores desde a fase 4. A vacinação contra covid-19 iniciou-se na fase 4 para maiores de 18 anos e, na fase 5, para maiores de 12 anos, mostrando forte impacto na redução da hospitalização e óbitos da população pediátrica.

A taxa de mortalidade hospitalar na população pediátrica no Brasil foi maior nas fases 1, 2 e 3, acompanhando, assim, os fenômenos de disseminação/interiorização do vírus no país, início da segunda onda e início da terceira onda, respectivamente.

Ressalta-se a importância de os indivíduos pré-escolares e lactentes serem vacinados, tendo em vista a aprovação e liberação, pela Anvisa, da vacinação de crianças maiores de 6 meses, em 16 de setembro de 2022, a fim de minimizar os efeitos da pandemia no grupo etário de crianças e adolescentes e realizar a proteção indireta dos lactentes menores de 6 meses ainda não contemplados com a vacina.
